# Recent progress in Fe- and Ru-based full-Heusler bulk thermoelectrics

**DOI:** 10.1080/14686996.2025.2517537

**Published:** 2025-06-11

**Authors:** F. Garmroudi, M. Parzer, T. Mori, E. Bauer

**Affiliations:** a Institute of Solid State Physics, TU Wien, Wien, Austria; b Materials Physics Applications - Quantum, Los Alamos National Laboratory, Los Alamos, USA; c International Center for Materials Nanoarchitectonics (WPI-MANA), National Institute for Materials Science (NIMS), Tsukuba, Japan

**Keywords:** Full-Heusler materials, thermoelectricity, electronic and thermal transport, DFT calculations

## Abstract

Full-Heusler compounds represent a rich and diverse class of functional materials, covering a large compositional phase space. Representatives with 24 valence electrons are commonly semimetals or narrow-gap semiconductors as per the Slater-Pauling rule and are thus considered as thermoelectric materials, especially for room-temperature applications. Research on the archetypal thermoelectric full-Heusler compound Fe_2_VAl began over two decades ago, and since then, significant progress has been made in enhancing its thermoelectric performance. Advances have been achieved through various intrinsic and extrinsic substitutions, grain boundary engineering and other optimization strategies. Here, recent advancements are reviewed, challenges for the further development of competitive full-Heusler thermoelectrics are identified, and novel routes and concepts are highlighted that could make these materials viable for energy harvesting and cooling applications near room-temperature.

## IMPACT STATEMENT

Full-Heusler compounds have emerged as important functional materials. Here, we review latest achievements for Fe- and Ru-based full-Heusler narrow-gap semiconductors as thermoelectric materials. Recent years have seen tremendous progress when it comes to enhancing the efficiency of these materials, reaching now values of the figure of merit zT up to 0.4–0.5 at room temperature. Our review will be valuable to the thermoelectric community seeking to employ new materials for near room-temperature applications.

## GRAPHICAL ABSTRACT

**Figure:**
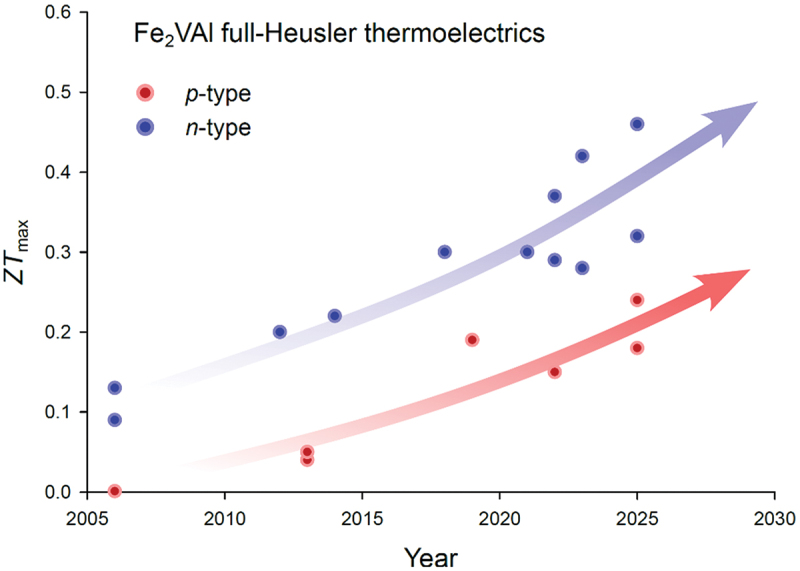

## 1. Introduction

Thermoelectricity is a steadily growing field in solid-state physics, computational materials science, chemistry and various branches of engineering. It seamlessly integrates fundamental research with application-driven developments and holds great potential for energy saving and recovery across multiple domains. Industrial processes, technical devices and everyday appliances inevitably lose part of their initial energy input as waste heat, rather than fully converting it into useful mechanical, thermodynamic or electrical work.

To recover – at least – a fraction of these losses would be invaluable with respect to economy as well as ecology. Thermoelectric generators can convert such waste heat into electricity, albeit with a limited efficiency, which generally remains below 10% today. The efficiency, η, of thermoelectric devices depends on the temperature range (Carnot efficiency) and is governed by the specific active thermoelectric materials via the temperature-dependent dimensionless figure of merit, ZT [1,2]. The latter combines the Seebeck coefficient S, the electrical resistivity ρ and the thermal conductivity κ – comprising the electronic ($\kappa_{\mathrm{el}}$) and lattice contributions ($\kappa_{\mathrm{ph}}$) – as $\mathrm{ZT}=TS^{2}/(\rho \kappa )$. Typically, ZT>1 is considered as a benchmark for useful applications. Since, however, the physical quantities constituting ZT are interdependent, increasing ZT is difficult, requiring sophisticated concepts and strategies.

Currently, the only materials used in commercially available thermoelectric devices and generators are alloys based on Bi_2_Te_3_. Known since the 1950s [3–5], these materials exhibit their highest performance at temperatures around $100{\;}^{\circ }$C. While Bi_2_Te_3_ is, in many aspects, an almost perfect thermoelectric material, it has notable drawbacks, including limited mechanical and chemical stability, as well as concerns regarding the scarcity of Te. Addressing these limitations is crucial for developing alternative thermoelectric materials that can support broader and more sustainable applications. Criteria are, besides a reasonably large value of ZT, the availability of the respective elements, the price and the long-term ability to supply. Additionally, workability and long-term stability of the materials under thermal gradients play a crucial role in applications. In this context, full-Heusler compounds and alloys based on $Fe_{2}\mathrm{VAl}$ are promising candidates to replace $Bi_{2}Te_{3}$ alloys in the long run.

Here, we review recent progress in the development of Fe- and Ru-based full-Heusler compounds and outline strategies on how to improve their thermoelectric figure of merit around room temperature. Compared to their widely studied half-Heusler relatives, which are considered for mid- and high-temperature waste heat recovery, full-Heusler alloys are attractive for near room-temperature applications due to their smaller band gaps and other unique band structure features as discussed in this review. This contribution to the Science and Technology of Advanced Materials topic ‘Advanced Thermoelectric Materials and Devices for Energy Harvesting’ is organized as follows: First, we provide an overview of the family of Heusler compounds and their crystal structures. Next, we discuss chemical design rules and electronic structure features that make Heusler compounds promising thermoelectric materials. Thereafter, we introduce Fe_2_VAl as the archetypal full-Heusler thermoelectric material and identify challenges that currently limit the applicability of these systems as well as recent progress that resulted in considerable enhancement of the thermoelectric performance. Finally, we review and present an outlook on Ru-based full-Heusler compounds as potential thermoelectric materials and directions that can be explored for the discovery of new Heusler thermoelectrics.

Heusler compounds and alloys were introduced in 1905 by Friedrich Heusler [6]. He demonstrated that a combination of non-ferromagnetic elements (Cu, Mn, Al) in an alloy yields a permanent magnetic material. Early X-ray diffraction studies by Young in 1923 [7] and Harang in 1927 [8] revealed , essentially, a cubic crystal structure with either bcc or fcc habit. Otto Heusler in 1934 [9] as well as Bradley and Rodgers [10] figured out the relevant details of the nowadays accepted crystal structure (space group: Fm-3 m, No.225, lattice parameter a = 0.5935 nm). Since then, hundreds of Heusler systems have been found and studied in detail. Many of them are successfully implemented in technical applications like in the field of spintronics, or involving qualities like shape memory properties or ferromagnetism. Besides, a large variety of ground states are present in Heusler systems, including superconductivity, magnetic skyrmions, Weyl semimetals, spin glass or magnetocaloric effects, half-metallicity, giant anomalous Nernst coefficients, as well as thermoelectricity. The widespread variation of physical properties is a consequence of the electronic structure of these $X_{2}\mathrm{YZ}$ full-Heusler systems, being intimately related to the respective cubic crystal structure. Depending on the elements involved in the $X_{2}\mathrm{YZ}$ materials, a semiconducting or metallic behavior may result as a consequence of the electronic band structure in the vicinity of the Fermi energy. In fact, a simple criterion can determine whether a given Heusler system is metallic or semiconducting, i.e. the so-called valence electron count $N_{\mathrm{VEC}}$ (see e.g. Ref [11]). If this count is 24, full-Heusler materials are expected to exhibit a gap in the electronic density of states around the Fermi energy. If, however, NVEC < x>24, bands are crossing the Fermi energy; thus, the system should behave metallic. Hence, thermoelectric materials based on full-Heusler systems should have their best performance for $N_{\mathrm{VEC}}\approx 24$. Examples for such materials are $Fe_{2}\mathrm{VAl}$ or $Fe_{2}\mathrm{TiSi}$. In addition, $N_{\mathrm{VEC}}$ reveals information about the magnetic state of Heusler compounds. The magnitude of the resulting permanent magnetic moment m follows the famous Slater-Pauling rule [12,13] and can be obtained from $m=(N_{\mathrm{VEC}}-24)\;\mu_{B}/f.u.$. Although $Fe_{2}\mathrm{VAl}$ or $Fe_{2}\mathrm{TiSi}$ contain 50% iron each, neither system exhibits magnetic ordering, in agreement with the Slater-Pauling predictions. Again, if NVEC < x>24, a permanent magnetic moment should develop and long-range magnetic ordering should be present. Examples following this rule and exhibiting magnetic order are, e.g., the archetypal compound Cu_2_MnAl, various Co- and Mn-based systems, such as Co_2_MnAl [14], Co_2_FeSi [15], Mn2VAl [16] and countless other full-Heusler materials. In fact, Co_2_FeSi ($N_{\mathrm{VEC}}=30$) orders magnetically as high as $T_{C}\approx 1100$ K and exhibits an ordered moment $m=6\;\mu_{B}$, in perfect agreement with the Slater-Pauling rule [15]. More recently, however, examples emerged that deviate from the Slater-Pauling rule. The implications of these deviations, particularly regarding key thermoelectric properties, are discussed later in this work.

## 2. Crystal structures of Heusler compounds

Ideal full-Heusler compounds of the type $X_{2}\mathrm{YZ}$ are found to crystallize in the cubic structure type $Cu_{2}\mathrm{MnAl}$ with space group Fm-3 m (no. 225); 4 formula units constitute the crystalline unit cell. X and Y are transition metals or rare earth elements, and Z is typically a main group element, e.g. from groups IIIA or IVA. In general, the atomic number $Z^{*}$ is larger for the X element, i.e. $Z_{X}^{*}>Z_{Y}^{*}$. Members of this family are numerous, as e.g. $Co_{2}\mathrm{MnAl}$, $Co_{2}\mathrm{TiAl}$, $Fe_{2}\mathrm{VAl}$ or $Ru_{2}\mathrm{TiSi}$, just to name a few. The unit cell of the crystal structure of $X_{2}\mathrm{YZ}$ is constituted by four interpenetrating fcc sublattices; two of them are equally occupied by X atoms. The local positions of the various elements (Wyckoff positions) in the unit cell are 8c (1/4, 1/4, 1/4) for X, 4a (0, 0, 0) for Y and 4b (1/2, 1/2, 1/2) for Z. At a local view, one of the X elements, together with the Z element form a zincblende sublattice; the second X element occupies the thus formed tetrahedral sites, while the Y element is located at the octahedral holes [17]. Depending on $N_{\mathrm{VEC}}$, a broad variety of ground states have been proposed theoretically and confirmed experimentally. If one of the X elements in $X_{2}\mathrm{YZ}$ is exchanged by another one, equiatomic quaternary Heusler compounds, $X{\;}^{\prime }X{\;}^{\prime }{\;}^{\prime }\mathrm{YZ}$, are formed. Typical examples here are CoFeCrAs, CoMnVAs, CoFeCrZ (Z = Si, As, Sb) and CoFeCrX (X = Si, Ge) [18–20]. There are several examples of so-called inverse full-Heusler compounds, where $Z_{X}^{*}<Z_{Y}^{*}$. Examples of this specific subgroup are e.g. $Fe_{2}\mathrm{RhSi}$, $Mn_{2}\mathrm{FeSi}$, $Mn_{2}\mathrm{CoSn}$ or $Fe_{2}\mathrm{RuGe}$. Inverse Heusler structures are formed by interchanging the X and Y positions, which, however, reveals a different type of crystal structure. Since X is more positive than Y, a rocksalt structure is created. X atoms are placed on the Wyckoff positions 4a (0, 0, 0) and 4d (3/4, 3/4, 3/4), Y elements at 4b (1/2, 1/2, 1/2), and Z elements are found at 4c (1/4, 1/4, 1/4). The prototype of this structure is $C\mathrm{uH}g_{2}\mathrm{Ti}$, space group F-43 m (no. 216). Another subgroup of full-Heusler compounds are anti-full-Heusler systems $Z_{2}\mathrm{XY}$, like $Ga_{2}\mathrm{MnCo}$, $Ga_{2}\mathrm{MnPd}$, $Al_{2}\mathrm{MnCo}$ or $Al_{2}\mathrm{MnPd}$ [21–24]. If one of the sublattices in $X_{2}\mathrm{YZ}$ is left free, half-Heusler compounds, XYZ, are originated, crystallizing in a non-centrosymmetric cubic structure (space group no. 216, F43m). The X and the Z elements are forming a zincblende structure, where the octahedral lattice sites are filled up by the Y elements. The Wyckoff positions of X, Y and Z are 4a (0, 0, 0), 4b (1/2, 1/2, 1/2) and 4c (1/4, 1/4, 1/4), respectively [17]. Typical members are, e.g., (Ti,Zr)NiSn, NbRhSn or (Nb,Ta)FeSb. These ternary half-Heusler compounds are the basis for high-performance thermoelectric materials with ZT>1.

Besides the dominating cubic crystal structure of Heusler compounds, lattice distortions can lead to tetragonal Heusler systems, which are found frequently in $XMn_{2}Z$ systems. Faleev et al. [25] proposed that such tetragonal distortions are originated by the peak-and-valley character of the density of states (DOS) of these compounds in the cubic phase, in conjunction with a smooth shift of the peaky DOS structure relative to the Fermi energy, when valence electrons are added to the system. Some of the Heusler compounds undergo martensitic phase transitions towards monoclinic or tetragonal phases. Examples in this respect are e.g. Co_2_Ti_1-x_Fe_x_As [26] or Ni_2_MnGa, exhibiting a phase transition from a cubic to a tetragonal system around 200 K [27]. Another variant of Heusler compounds exhibits a hexagonal crystal structure, space group P6${\;}_{3}$/mmc, no. 194, with the prototypic $Ni_{3}\mathrm{Sn}$ structure. A typical member here is $Fe_{2}\mathrm{MnGe}$; there, both Fe and Mn occupy the 6 h site (0.1667, 0.3333, 0.25) with occupations 2/3 and 1/3, respectively. Ge is located in the unit cell at 2c (0.3333, 0.6667, 0.25) [28]. More specific details regarding crystal structure features of the various groups and sub-groups of Heusler systems have been summarized in detail in recent review articles, e.g. in the seminal paper of Graf et al. [17] as well as in Refs [25,29,30].

**Figure 1.**
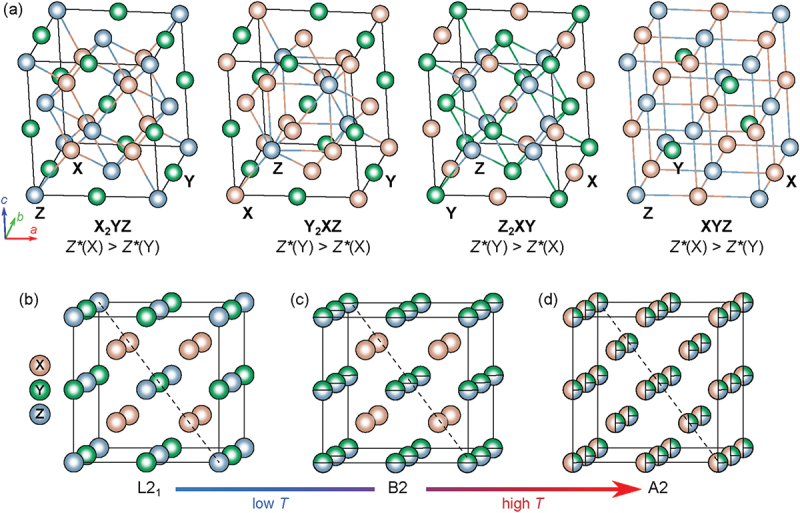
(a) Crystal structures for various phases of Heusler compounds; from left to right: full-Heusler compound $X_{2}\mathrm{YZ}$, inverse-Heusler compound $Y_{2}\mathrm{XZ}$, anti-Heusler compound $Z_{2}\mathrm{XY}$ and half-Heusler compound XYZ. Temperature- or mechanically induced order–disorder transitions frequently occur in the family of Heusler compounds and can change the crystal symmetry, e.g. from the (b) face-centered cubic L2${\;}_{1}$ structure to the body-centered cubic (c) partly disorder B2 and (d) fully disordered A2 structure.

A sketch of the different crystal structures is presented in Figure 1. There, the X atoms are displayed in light orange, the Y atoms in light green and the Z atoms in light blue. As was shown in detail for $Fe_{2}\mathrm{VAl}$ by DFT calculations in terms of the so-called Bader approach, there is a distinct charge transfer of 0.75 electronic charges to each Fe atom from Al (1.03) and V (0.48) [31]. Thus, bonds are drawn throughout Figure 1(a) from the X element towards the Z element. At this point, we also note that charge transfer and the lack thereof for certain defects gives rise to the existence of ionized states in $Fe_{2}\mathrm{VAl}$; hence, very distinct scattering processes can happen [32], modifying temperature dependencies of physical properties, which are normally dictated by the scattering of charge carriers off acoustic phonons or uncharged impurities. Heusler compounds, specifically full-Heusler systems, are prone to anti-site occupations, i.e. the various constituent elements do not occupy their distinct sites, rather, atoms of a specific type interchange their local sites with other ones on another site. For instance, atoms on the 4a and 4b site frequently exchange their position, thus creating disorder on both sublattices. The probability for such transpositions depends on the formation enthalpy of such a process as well as on the gain in configurational entropy. The latter increases linearly with temperature, which makes antisite disorder more favorable and likely at elevated temperatures, as discussed below. Employing DFT calculations, Bandaru and Jund [33] have derived formation enthalpies of different defects in $Fe_{2}\mathrm{VAl}$, finding that the most likely transpositions are antisite defects like $Al_{V}$, $Al_{\mathrm{Fe}}$ and $V_{\mathrm{Al}}$, significantly more energetically favorable than defects such as vacancies or interstitials. Antisite effects not only cause an increase of crystallographic disorder or modifications in the electronic density of states, but they can also entirely change the magnetic state, and a non-magnetic material can become magnetic. This was demonstrated both theoretically [33] and experimentally [34,35] on $Fe_{2}\mathrm{VAl}$. Long-range magnetic order is established in $Fe_{2}\mathrm{VAl}$ for V vacancies as well as $Fe_{V}$ and $V_{\mathrm{Fe}}$ antisite defects. By quenching $Fe_{2}\mathrm{VAl}$ from different heating temperatures, various states of disorder can be frozen, and physical properties can be measured straightforwardly. As a result, different overall magnetic moments have been obtained, as a consequence of the different forms of vacancies or antisite transpositions and p-type conduction switches to n-type [36]. These frozen structures mirror structural phase transitions of $Fe_{2}\mathrm{VAl}$ from A2 (at 1190 ${\;}^{\circ }$C) to B2 (at 1080 ${\;}^{\circ }$C) and L2${\;}_{1}$ down to room temperatures [37]. The increase of disorder due to a growing number of antisite occupations, as it occurs in the thermoelectric full-Heusler material $Fe_{2}\mathrm{VAl}$, is sketched in Figure 1(b–d). While the thoroughly ordered full-Heusler system exhibits the classical Heusler structure L2${\;}_{1}$ ($Cu_{2}\mathrm{MnAl}$-type, space group Fm-3 m, no. 225), the B2 structure (space group: Pm-3 m, no. 221) is characterized by anti-site occupations of Al and V. If eventually all elements interchange their original positions, the A2-type (space group: Im-3 m, no. 229) is approached, characterized by a fully disordered body-centered cubic structure.

## 3. Selecting promising thermoelectric materials from full-Heuslers

Since thermoelectric generators are thermodynamic machines, they are following Carnot’s principles, i.e. the temperature range of operation is of primary importance for the thermoelectric performance of a certain thermoelectric device. Accordingly, the largest average ZT values of a distinct material need to be inside that temperature range. An initial designing principle follows $E_{g}\approx 10k_{B}T_{\mathrm{work}}$, where $E_{g}$ is the band gap in the electronic density of states at the Fermi energy, $k_{B}$ is the Boltzmann constant and $T_{\mathrm{work}}$ is the relevant temperature of device operation. Because the intention of the present review is focused on well performing materials in the 100 ${\;}^{\circ }$C range, the relevant gap in the DOS of typical materials should be a few hundred meV. Several full-Heusler $X_{2}\mathrm{YZ}$ materials can be conceived, matching this criterion, either by an already existing gap of suitable width or by appropriate isoelectronic or non-isoelectronic substitutions on the various lattice sites of the $X_{2}\mathrm{YZ}$ crystalline unit cell. Heavier isoelectronic elements are expected to increase the gap in the electronic density of states, while aliovalent substitution elements shift the position of the Fermi energy in an otherwise unchanged density of states (rigid band model). Ternary full-Heusler compounds with 24 valence electrons, like Fe_2_VAl, Fe_2_VGa, Fe_2_TiSi, Fe_2_TiSn, Ru_2_NbGa, Ru_2_TaAl, Ru_2_TiSi, Ru_2_TiGe, etc., are found to exhibit such relatively narrow gaps in the DOS near the Fermi energy. In addition, they display low-dimensional Fermi surface features owing to non-parabolic bands that are flat in certain directions of the first Brillouin zone but highly dispersive in others, creating a strongly energy-dependent transport distribution function Σ(E), which can be derived from Boltzmann transport theory and, in the isotropic case, is formally defined as

(1) $$\Sigma (E)=\sum_{i=1}^{N}\frac{1}{{\left(2\pi \right)}^{3}}\int \tau_{i,k}v_{i,k}v_{i,k}\delta (E-E_{i,k})d^{3}k,$$

simplifying for spherical Fermi surfaces to $\Sigma (E)=D(E)v^{2}(E)\tau (E)$. D(E), $v^{2}(E)$ and τ(E) are the electronic density of states, the squared group velocity of charge carriers and the energy-dependent carrier relaxation time, respectively. Σ(E) determines all electronic transport properties of a material, including the power factor

(2) $$\sigma S^{2}=\frac{{\left[\int_{-\infty }^{\infty }\Sigma (E)\frac{E-\mu }{T}\left(\frac{-\partial f_{0}(E)}{\mathrm{\partial E}}\right)\mathrm{dE}\right]}^{2}}{\int_{-\infty }^{\infty }\Sigma (E)\left(\frac{-\partial f_{0}(E)}{\mathrm{\partial E}}\right)\mathrm{dE}\;},$$

where σ is the electrical conductivity, S the Seebeck coefficient, μ the chemical potential and $f_{0}$ the Fermi-Dirac distribution function. As originally pointed out by Mahan and Sofo [38], well performing thermoelectric materials should exhibit a strong energy dependence of Σ(E), the ideal form of which would be a delta-distribution function $\Sigma (E)\;∼\;\delta (E-E_{0})$, with μ situated a few $k_{B}T$ away from $E_{0}$. This can be achieved by (i) a distribution of charge carrier energies as narrow as possible and (ii) high carrier velocities in the direction of the applied electric field [39], which requires, for the former, flat energy bands, but for the latter, strongly dispersive bands near the Fermi energy. Such bands can, in fact, be found in various full-Heusler systems, like in $Fe_{2}\mathrm{VAl}$ (see Figure 4) or Fe_2_TiSi, and other isovalent Fe_2_YZ-based members. There, Fe-$e_{g}$ states form a very flat conduction electron band, with almost no dispersion along Γ – X but a strong dispersion in other Brillouin zone directions. As mentioned previously, simple chemical rules of electron counting, such as the Slater-Pauling rule, allow one to identify systems where $E_{F}$ falls close to such a band edge. For full-Heusler compounds, semiconducting ground states are usually realized when the compound has a total of 24 valence electrons, while half-Heusler relatives require 18 valence electrons. Almost all promising thermoelectric Heusler materials are valence-balanced systems with an average valence electron count (VEC) of six valence electrons per atom.

## 4. The full-Heusler compound Fe_2_VAl

In the realm of thermoelectrics, full-Heusler systems based on Fe_2_VAl undeniably stand out as the most extensively studied members within the full-Heusler family. Subsequently, this section provides a concise overview of previous experimental and theoretical studies. Following that, a discussion is undertaken regarding the most critical research gaps that have been effectively addressed in recent years and need to be addressed further to make this material competitive to state-of-the-art Bi_2_Te_3_ semiconductors.

### 4.1. Experimental considerations

Fe_2_VAl-based systems have gained considerable interest from both a fundamental point of view and also for applications such as thermoelectrics, owing to their peculiar electronic structure, which has been a subject of extensive debate. In particular, Nishino et al. already observed a semiconductor-like transport behavior over two decades ago [42]. The Arrhenius-type behavior of the electrical resistivity at high temperatures $\rho (T)\propto \exp(E_{g}/(2k_{B}T))$ suggests a finite band gap of 0.1 eV, comparable to that of the correlated narrow-gap semiconductor FeSi. Photoelectron spectroscopy measurements by Soda et al. [43], on the other hand, showed a clear Fermi cutoff, indicating a rather metallic ground state DOS. Together with a moderately enhanced effective mass derived from specific heat measurements at low temperatures, the question of whether Fe_2_VAl could be a possible candidate for a 3d heavy fermion compound was raised by Nishino et al. in 1997 [42]. In response to these assertions, various experimental studies, including optical conductivity [44] and nuclear magnetic resonance [45,46], were carried out. These measurements falsified the previously suggested scenario and instead proposed a pseudogap of 0.2 eV or larger (compare Table 1). In this context, the density of states at the Fermi energy $D(E_{F})$ remains finite but small, accounting for the semiconductor-like characteristics observed. These interpretations align with early theoretical studies by Weinert and Watson in 1998 that predicted hybridization-induced band gaps in various transition metal aluminides, including Fe_2_VAl [47]. Moreover, the 3d heavy fermion scenario was further excluded by field-dependent specific heat measurements, performed by Lue et al. in 1999 [61], who instead invoked strong spin fluctuations as the primary cause for the enhanced effective masses, inferred from heat capacity measurements [42].

**Table 1.** Energy band gap $E_{g}$ for stoichiometric Fe_2_VAl from the literature [31,32,42,44,45,47,48–53], derived by various *ab initio* methods [54–59] and experiments. Large negative and positive values of $E_{g}$ are possible, depending on the treatment of exchange correlation effects which are used for the band structure calculations.

	Method	Band gap	Reference
DFT:	LDA (FLASTO)	−0.2 eV	Weinert, Watson [47]
	LSDA, LAPW	−0.2 eV	Singh, Mazin [48]
	PBEsol	−0.2 eV	Shastri et al. [49]
	SCAN	−0.16 eV	Shastri et al. [49]
	GGA-PBE	−0.1 eV	Bandaru et al. [53]
	GGA-EV	−0.06 eV	Do et al. [52]
	GGA$+U_{\mathrm{Fe}-d}$	−0.0092 eV	Hinterleitner et al. [31]
	QSGW	0.21 eV	Tomzcak [159]
	mBJ	0.22 eV	Shastri et al. [49]
	B1WC${\;}_{\alpha =0.16}$ eV	0.34 eV	Bilc et al. [50]
	GGA$\;+\;U_{\mathrm{Fe}-d,V-d}$	0.55 eV	Do et al. [52]
	PBE0	0.58 eV	Do et al. [52]
	B1WC${\;}_{\alpha =0.25}$	0.62 eV	Do et al. [52]
	HSE06	1.2 eV	Lee et al. [60]
Experiment:	Seebeck \amp resistivity	−0.12 eV	Garmroudi et al. [32]
	Seebeck coefficient	0.002 eV	Hinterleitner et al. [31]
	Seebeck coefficient	0.02−0.04 eV	Anand et al. [51]
	Seebeck coefficient	0.002 eV	Hinterleitner et al. [31]
	Electrical resistivity	0.1 eV	Nishino et al. [42]
	Photoconductivity	0.1−0.2 eV	Okamura et al. [44]
	NMR Knight shift	0.22 eV	Lue et al. [45]
	Spin relaxation rate	0.27 eV	Lue et al. [45]

**Figure 2.**
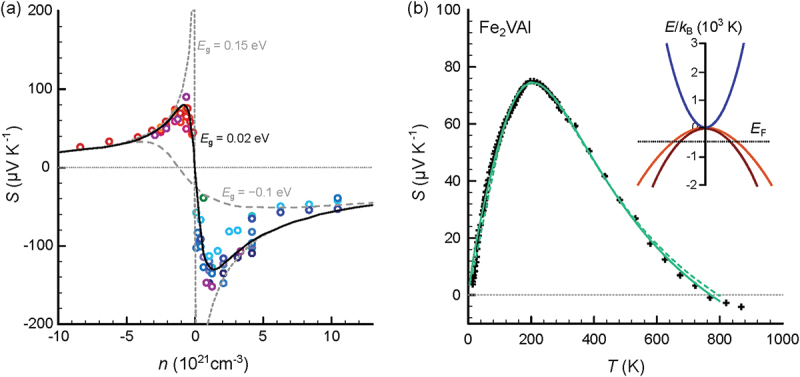
(a) Seebeck coefficient of Fe_2_VAl-based compounds as a function of the carrier concentration for various aliovalent substitution studies [63,64,66–78]. Solid, dashed and dotted lines are model results, employing a parabolic two-band model with dominant acoustic phonon scattering. Different values of the band gap $E_{g}$ are compared. While a negative band overlap (semimetal) $E_{g}=-0.1\;$eV fails to describe the experimental data, the data can be well described assuming a finite band gap of $E_{g}=0.02\;$eV. Data are taken from ref [51]. (b) Temperature-dependent Seebeck coefficient of Fe_2_VAl. Solid and dashed lines represent least-squares fits to the experimental data, employing a triple and two-parabolic band model, respectively. The resulting band structure (inset) features a tiny positive band gap ≈2meV between valence and conduction bands and an almost degenerate set of valence bands, consistent with density functional theory calculations employing GGA + U functionals.

From a perspective of thermoelectric measurements, the Seebeck coefficient reaches sizable values up to 70μV K${\;}^{-1}$ in the undoped material [31] and even larger absolute values up to 180μV K${\;}^{-1}$ in n-doped and 110μV K${\;}^{-1}$ in p-doped samples [62] with a peak close to room temperature, indicative of narrow-gap semiconductors [31,63–65]. Indeed, when modeling the temperature-dependent Seebeck coefficient from 4 to 800K within a parabolic two- or three-band framework, a tiny positive band gap ≈2meV is obtained for the undoped compound (see Figure 2(a)). Furthermore, Anand et al. modeled the carrier-concentration dependence of the Seebeck coefficient for various Fe_2_VAl-based samples with aliovalent substitution (see Figure 2(b)) and found an optimal band gap around $E_{g}=0.02\;$eV to describe the data. These fitted values, however, depend on the scattering parameter, which is initially assumed/set when modeling temperature-dependent transport properties. Therefore, fitting results can give varying values in the range −0.15eV up to +0.002 eV, depending on the choice of relevant model parameters [32].

### 4.2. Theoretical considerations

**Figure 3.**
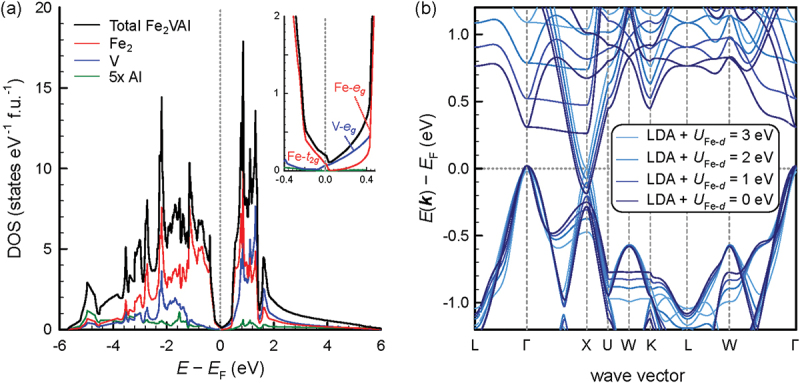
(a) Calculated (GGA-PBE) electronic density of states (DOS) of Fe_2_VAl with atom-decomposed contributions. Inset shows a close-up of the DOS around $E_{F}$, relevant for electronic transport. It can be seen that the valence band consists mostly of Fe $t_{2g}$ states while the conduction band crossing $E_{F}$ has mainly V $e_{g}$ orbital character. The rather localized states ≈0.4eV above $E_{F}$ originate from Fe $e_{g}$ orbitals. (b) and (c) shows the band structure of Fe2VAl considering additional on-site Coulomb repulsion for Fe d orbitals and V d orbitals in the DFT calculations (LDA +U). An opening of the gap is apparent, driven by the Coulomb repulsion among Fe or V d states. (d) Temperature-dependent Seebeck coefficient of Fe_2_VAl. Black crosses are experimental data; solid and dashed lines are theoretical predictions, calculated from the band structure in the constant relaxation time approximation.

Figure 3(a) shows the density of states of Fe_2_VAl with the respective atom-decomposed partial densities of states. It can be seen that Fe states give the largest contribution to the overall DOS, whereas Al has a minimal contribution in the energy range −6 to 3 eV. Vanadium has a sizable contribution to the DOS above the Fermi energy as the conduction band dangling into the pseudogap (*X* band) has mainly V $e_{g}$ orbital character. Standard density functional theory (DFT) calculations in the local density approximation (LDA) or general gradient approximation (GGA) reveal a large negative indirect band overlap at different high-symmetry points in the reciprocal space (−0.2 to −0.1 eV) [47–49,79]. Simple exchange correlation functionals, such as LDA- and GGA-type functionals, thus cannot explain the semiconductor-like transport behavior and large Seebeck coefficients found experimentally. Considering hybrid functionals with different admixture parameters or enhanced correlation effects in terms of an effective onsite Coulomb repulsion, the band gap can be increased drastically from −0.2 up to 1.2 eV with a large variance of possible values in between, depending on the chosen functional (see Table 1). It was shown that corrections to the DFT calculations in terms of the single-particle Green’s function and screened Coulomb interaction (GW corrections) can lead to more consistent results among the different functionals [60]. Hinterleitner et al. investigated the effect of introducing an effective on-site Coulomb repulsion among Fe d orbitals $U_{\mathrm{eff}}=U-J$, where U is the Coulomb repulsion and J the exchange interaction. A large number of $U_{\mathrm{eff}}$ values were screened, and the temperature-dependent electronic transport properties (Seebeck coefficient, electrical resistivity) were calculated from the resulting band structures in the constant relaxation time approximation. By comparing their theoretical results to experimental data, a recommended value of $U_{\mathrm{eff}}=2.145\;$eV for the Fe d orbitals was determined by the authors, resulting in a tiny negative band overlap $E_{g}=E_{X}-E_{\Gamma }=-0.009\;$eV when calculating the band structure using this set of parameters. Figure 3(b) compares the band structure of Fe_2_VAl for different values of the on-site Coulomb repulsion term taken into account for the Fe-d orbitals. It can be seen that the band gap expands as the Fe $e_{g}$ states are pushed towards higher energies, whereas Fe-dominated $t_{2g}$ states are pushed towards lower energies. Interestingly, even the V-$e_{g}$ conduction states at X are affected and slightly raised in energy.

Bilc and Ghosez [50], Venkatesh et al. [80] as well as Bandaru and Jund [33] have pointed out that antisite defects can result in significant modifications of the electronic structure. Without knowing the exact contribution from defects and their local interactions in real samples, it has not been possible to derive a precise assessment of the intrinsic electronic structure so far. The same holds true for electronic correlation effects which are set as additional input parameters and hardly calculable ab initio. Recently, claims such as a temperature-induced band gap increase stemming from thermal expansion and thermally activated V/Al inversion defects have been invoked by Berche et al. [81] in order to justify the disparity between metallic low-temperature photoemission spectroscopy data and semiconductor-like high-temperature transport properties. These results, however, are based upon the assumption that V/Al inversion defects form clusters around Fe atoms. To this end, it is important to highlight that the authors overlooked the fact that, at elevated temperatures, a considerable increase in the configurational entropy term contributes significantly to the Gibbs enthalpy, favoring a disordered arrangement of defects and decreasing the likelihood of an ordered cluster configuration.

Summarizing, there have been ample theoretical attempts to explain experimental observations: (i) A semimetallic band structure with a vanishingly small band overlap [31] (ii) a wide-gap band structure with impurity in-gap states induced by antisite disorder [50], (iii) a semimetallic band structure with a temperature-induced band gap opening due to thermal expansion and antisite defects [81], as well as more exotic interpretations such as (iv) a metallic band structure incorporating dynamical correlation effects [82]. To conclude this section, it is crucial to emphasize that engaging in a meaningful discussion and theoretical comparison with experimental results of nominally stoichiometric Fe_2_VAl is futile without accounting for inevitable antisite defects and their critical role in altering electronic and magnetic properties in manifold aspects.

## 5. Challenges and progress in Fe_2_VAl thermoelectrics

### 5.1. Narrow band gap and bipolar conduction

As discussed in the previous section, although there is still ambiguity with respect to the precise value of the band gap, the full-Heusler compound Fe_2_VAl has typically been reported to feature an almost zero-gap or even semimetallic band structure with a small overlap of its valence and conduction bands. Still, the Seebeck coefficient can nonetheless reach sizable values in this system due to highly asymmetrical band features and a quasi-low-dimensional Fermi surface. Kato et al. first demonstrated that introducing Si at the Al site in Fe_2_VAl_1-x_Si_x_ effectively adjusts the charge carrier concentration and induces a rigid-band-like shift of $E_{F}$, already yielding substantial power factors PF≈ 5.5 mW m${\;}^{-1}$ K${\;}^{-2}$ [66], larger than those of Bi_2_Te_3_ systems and most other state-of-the-art semiconductors, while also maintaining large Seebeck coefficients up to $120-130\;mVK^{-1}$. Subsequent investigations revealed even higher power factors, reaching up to 6.7 mW m${\;}^{-1}$ K${\;}^{-2}$ [62] and 6.8 mW m${\;}^{-1}$ K${\;}^{-2}$ [83] in off-stoichiometric variations, such as Fe_2-x_V_1+x_Al_1-y_Si_y_ and Fe_2_V_1+x_Al_1-x_. Therefore, it is not surprising that over the course of the last years the primary focus of research has been on reducing the lattice thermal conductivity of Fe_2_VAl. However, to achieve substantial enhancements of the figure of merit ZT, it is crucial to further tune the band structure via band engineering as well, especially considering the rather low values of the Seebeck coefficients in p-type Fe_2_VAl-based full-Heuslers, which hinders progress in enhancing ZT. Indeed, even in the metallic limit, that is, if lattice-driven heat transport could be minimized such that heat transport is entirely dominated by electrons $\kappa_{\mathrm{el}}\gg \kappa_{\mathrm{ph}}$, the limit $\mathrm{ZT}\approx S^{2}/L$ – L being the Lorenz number – indicates that for p-type compounds, with S generally of the order of 100μV K${\;}^{-1}$ or less, it is impossible to obtain ZT values that are competitive with Bi_2_Te_3_ and its alloys with Sb_2_Te_3_. Anand et al. pointed out that an enhancement of the band gap is crucial to further optimize the TE performance of Fe_2_VAl-based thermoelectrics [51]. Certainly, an enhancement of the band gap increases the quality factor $\tilde{B}=\left(k_{B}/e^{2}\right)\left(\sigma_{E0}/\kappa_{\mathrm{ph}}\right)E_{g}$ in materials – $\sigma_{E0}$ being the electronic quality factor – where two bands actively contribute to TE transport [84]. Increasing $E_{g}$ not only allows for larger values of the Seebeck coefficient to be realized but also reduces bipolar thermal conductivity, whose contribution is significant in Fe_2_VAl-based systems at T>300K.

#### 5.1.1. V site substitutions to expand band gap

**Figure 4.**
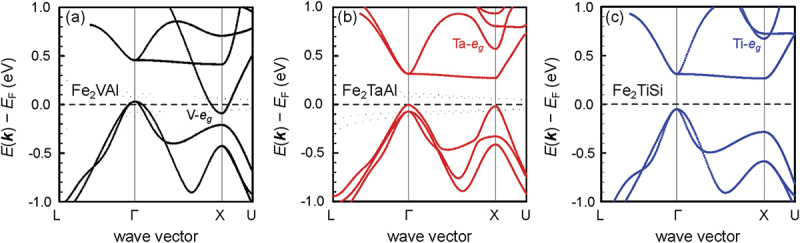
Electronic band structures at equilibrium volumes obtained from DFT calculations using GGA-PBE-type exchange correlation functionals. (a) Fe_2_VAl, (b) Fe_2_TaAl (with spin – orbit coupling). (c) Fe_2_TiSi. While Fe_2_VAl is a semimetal the latter two are semiconductors due to Ta- and Ti-$e_{g}$ states being higher in energy. Computational data were obtained by the authors in previous studies [40,41] and computational details are summarized in the respective references.

Since the pseudogap states dangling into the band gap and overlapping in energy are dominated by Fe-$t_{2g}$ states for the valence band (VB) and V-$e_{g}$ states for the conduction band (CB), rational substitutions at the Fe or V sublattices are crucial to expand the band gap. What has proven as an effective way of expanding the band gap and thereby increasing the Seebeck coefficient and overall performance are co-substitutions on the V sublattice [40,41]. Figure 4 compares the band structure near the Fermi level of the isovalent full-Heusler compounds Fe_2_VAl, Fe_2_TaAl and Fe_2_TiSi, obtained in terms of standard GGA-PBE exchange correlation functionals. While Fe_2_VAl displays a semimetallic band structure, the other two compounds are predicted to be semiconductors, as the Y site-dominated $e_{g}$ conduction bands are pushed towards higher energies. In the case of Fe_2_TaAl, one may assume that this results from the 5d states of Ta being located higher in energy than the 3d states of V. For Fe_2_TiSi, the lower nuclear atomic charge and reduced Coulomb attraction likely explain the higher lying $e_{g}$ orbitals in this compound and the resulting semiconducting ground state.

**Figure 5.**
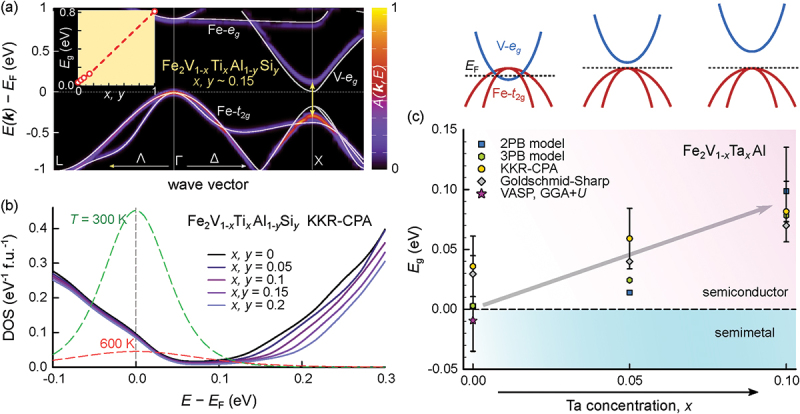
Band gap opening by partial Ta and Ti substitutions at the V site. (a) Unfolded band structure of partially substituted Fe_2_V_1-x_Ti_x_Al_1-y_Si_y_ with x=y=0.15. Inset shows a continues increase of the band gap with increasing x,y. (b) Alloy-averaged densities of states of Fe_2_V_1-x_Ti_x_Al_1-y_Si_y_ for various concentrations of x,y. Green and red dashed lines show derivative of the Fermi-Dirac distribution at 300 and 600 K, respectively. (c) a continuous expansion of the band gap is also confirmed for partial Ta substitution in Fe_2_V_1-x_Ta_x_Al. Computational data and figures were reproduced from Refs [40,41].

**Figure 6.**
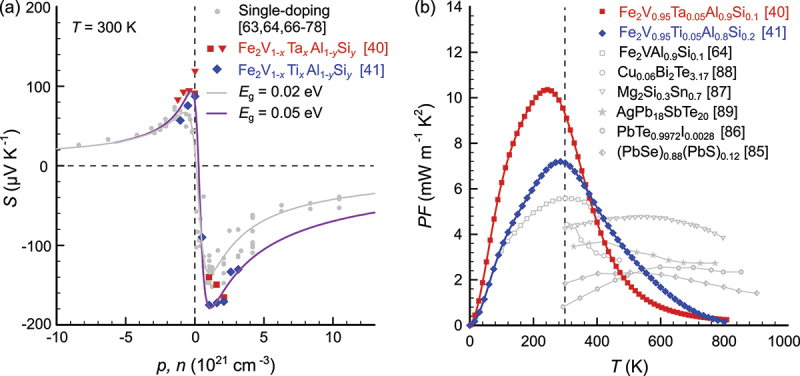
Thermoelectric performance enhancement by band gap engineering. (a) Pisarenko-style plot of room-temperature Seebeck coefficient versus chemical carrier doping concentration of single-doped Fe_2_VAl-based Heusler compounds [63,64,66–78] and co-substituted systems [40,41] based on Fe_2_V_1-x_Ta_x_Al_1-y_Si_y_ and Fe_2_V_1-x_Ti_x_Al_1-y_Si_y_, (b) temperature-dependent power factor of optimally doped co-substituted Heusler compounds with an enhanced band gap versus single-doped Fe_2_VAl_0.9_Si_0.1_ [64] and other state-of-the-art n-type thermoelectric materials [85–89].

An interesting and crucial question is, whether a continuous band gap opening can be achieved by partial substitution, especially since the stoichiometric compounds Fe_2_TaAl and Fe_2_TiSi are metastable and have so far not been successfully synthesized as single-phase materials in bulk form. Figure 5 shows the evolution of the electronic structure of Fe_2_VAl-based Heusler compounds upon partial substitution of V with Ti and Ta. Since Ti has one less valence electron than V, it is necessary to co-substitute Si at the Al site to balance the Fermi level. By a surplus of Ti doping or Si doping, the properties can be varied from p-type to n-type. Similarly, in the case of Ta substituted Heusler compounds, Ti and Si can be co-doped to enable a shift of the Fermi level further into the VB or CB. Figure 5(a) shows the unfolded electronic band structure of Fe_2_V_1-x_Ti_x_Al_1-y_Si_y_ (x,y≈0.15), obtained from DFT supercell calculations [41] using GGA-PBE functionals with an on-site Coulomb correction (GGA + U, $U_{\mathrm{Fe}-d}=2.145\;$eV). The white solid lines represent the band structure of stoichiometric pristine Fe_2_VAl using the same computational parameters. It can be clearly seen that even for rather small substitutions x,y, an increase of the band gap is still visible as the V-$e_{g}$-dominated band is shifted towards higher energies. The inset in Figure 5(a) highlights that there exists a continuous, almost linear increase of the band gap between the two Heusler compounds (Fe_2_VAl and Fe_2_TiSi). Consistent and qualitatively similar results are also found for DFT-based calculations using the framework of the Kohn-Korringa-Rostoker method and the coherent potential approximation (KKR-CPA), which accurately capture alloy disorder arising from the random substitution of Ti and Si atoms at the V and Al sublattices, respectively (see Figure 5(b)). The same picture holds true and has been confirmed experimentally also for partial Ta substitution in Fe_2_V_1-x_Ta_x_Al (see Figure 5(c)). Thus, it is possible to tune (expand) the band gap of Fe_2_VAl via partial substitution of Ta and Ti at the V site. As shown in Figure 6(a), this leads to significantly higher Seebeck coefficients, S>190μV K${\;}^{-1}$, which can be realized in these co-substituted systems. Consequently, ultrahigh values of the thermoelectric power factor, PF>10mW m${\;}^{-1}$ K${\;}^{-2}$ were achieved, which are more than two times larger than those of the best Bi_2_Te_3_-based systems (see Figure 6(b)). We note that these values constitute the record among n-type semiconductors, whereas even larger power factors are achieved in metallic systems with scattering-induced electron–hole asymmetry such as the intermediate valence Kondo system YbAl_3_ [90] and binary transition metal alloys [91]. The highest power factor of any material above room temperature is obtained in binary Ni_x_Au_1-x_ alloys, with $PF_{\max}\approx 34\;$mW m${\;}^{-1}$ K${\;}^{-2}$ at 560 K and an ultrahigh average $PF_{\mathrm{av}}\approx 30\;$mW m${\;}^{-1}$ K${\;}^{-2}$ in the temperature range 300–1100 K for x=0.1 [91].

#### 5.1.2. Off-stoichiometry and self-subsitution

Another approach to modify the electronic structure of Fe_2_VAl is self-substitution, where the compound is doped with its own constituents – Fe, V and Al. This approach offers advantages such as a high solubility limit for various substitution strategies and a straightforward synthesis process, as all three elements are inexpensive and easy to handle. Various studies on off-stoichiometry in Fe_2_VAl have explored different substitution variations, including Fe/V [62,92–97], V/Al [83,98–103], Fe/Al [96,104], as well as Fe, V/Al off-stoichiometry [67,92,105–110]. Notably, full solubility from V_3_Al to Fe_3_Al was reported in Fe_2+*x*_ V_1 – *x*_ Al [111], and an exceptionally high solubility limit of x=0.2 was found for Fe, V substitution with Al in Fe_2-2x_V_1-x_Al_1 + 3x_ [108,110]. Additionally, no solubility limits were observed for V/Al and Fe/Al substitutions up to 20% [83,104]. For thermoelectric applications, the self-substitution of Al with V, combined with V/Ti substitution, has shown promising results, achieving the highest TE performance in p-type Fe_2_VAl materials reported to date (ZT up to 0.24 at 400 K) [101,103,112]. This improvement can possibly be attributed to a slight band gap opening due to the off-stoichiometry and the V/Ti substitution, as described above. Following a related approach, Parzer et al. employed double-site substitution of V and Al atoms by Ti to expand the band gap, although decreasing the Al content ultimately results in a closure of the band gap following the transition from Fe_2_VAl to Fe_2_Ti_2_ [113]. Thus, a sweet spot exists, where $E_{g}$ and consequently S can be enhanced, leading to ZT≈0.2.

**Figure 7.**
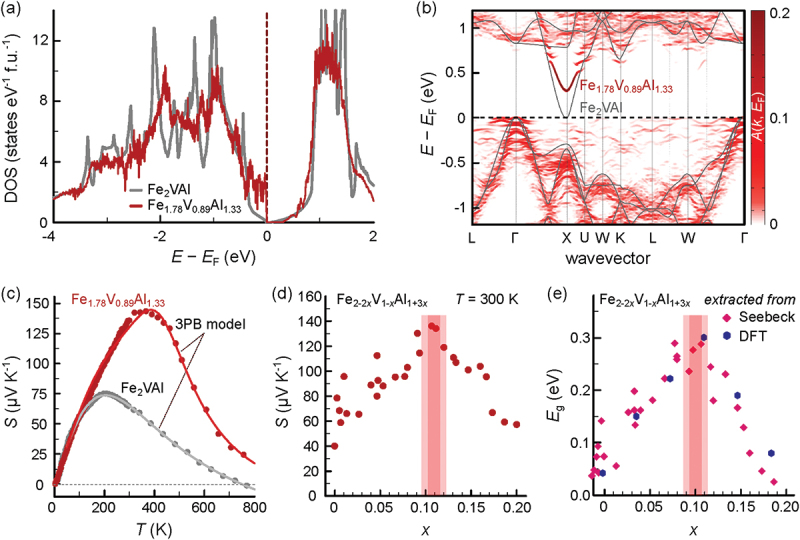
(a) Comparison of the densities of states of stoichiometric Fe_2_VAl with the Al-rich Fe_1.78_V_0.89_Al_1.33_ compound. (b) Comparison of the band structures of stoichiometric Fe_2_VAl with the Al-rich Fe_1.78_V_0.89_Al_1.33_ compound. For the off-stoichiometric calculation, a 108-atomic supercell was calculated, subsequently employing band unfolding. (c) Comparison of the temperature-dependent Seebeck coefficient of the two compounds. Clearly, the Al-substitution leads to a significant increase in S(T). (d) Seebeck coefficient at room temperature for multiple samples along the Fe_2-2x_V_1-x_Al_1 + 3x_ stoichiometry ranging from x=0−0.2. The red area highlights the samples that are close to the theoretical highlighted compound in panels (a-c), with x=0.11. (e) Extracted band gap for the same samples from both, temperature dependence of the experimental Seebeck coefficients (red diamonds), and DFT calculations (blue hexagons). The Seebeck coefficient follows a similar trend as the band gap, confirming the strong correlation between $E_{g}$ and S(T) in these compounds. Experimental and computational data were obtained by the authors in a previous study [110].

On the other hand, the simultaneous substitution of Fe and V with Al was found to induce more drastic changes in the electronic structure [108,110]. As highlighted in Figure 7(a,b) Al self-substitution leads to a significant flattening of the Fe-dominated valence band together with a substantial band gap opening reaching close to $E_{g}=0.3$ eV in Fe_1.78_V_0.89_Al_1.33_. This was traced back to a lack of d-d hybridization of the Al antisites with the surrounding V and Fe atoms. Consequently, the Seebeck coefficient is nearly doubled compared to stoichiometric Fe_2_VAl and its maximum shifts towards higher temperatures close to 400 K, as bipolar conduction is reduced (see Figure 7(c)). Figure 7(d,e) show the composition-dependent change in the Seebeck coefficient and the band gap $E_{g}$, extracted from S(T) via the three-band model [114] and from DFT supercell band structure calculations, analyzing multiple samples along the Fe_2-2x_V_1-x_Al_1 + 3x_ series for x=0−0.2 [110]. A continuous trend is observed with increasing Al substitution: up to 11% substitution of Fe and V atoms with Al, the band gap increases, while for higher x, it decreases. Interestingly, this band gap opening occurs despite substantially increasing disorder within the system and can be understood as a hybridization gap, as discussed in more detail in Refs [47,110]. Moreover, the similarity between S(x) and $E_{g}(x)$ underscores the importance of reducing the bipolar conduction, for instance, through band gap opening in Fe_2_VAl. Finally, the increased disorder leads to a reduced thermal conductivity in off-stoichiometric compounds, most drastically in the case of Fe_2-2x_V_1-x_Al_1 + 3x_ [108].

Besides introducing self-substitution/antisite defects through off-stoichiometry, Fe_2_VAl Heusler compounds have the unique ability to promote the formation of antisite defects even in their stoichiometric, pristine form upon changing the heat treatment. As discussed previously and highlighted in Figure 1(b–d), Fe_2_VAl undergoes two structural order–disorder transitions at high temperatures. First, at around 1080 ${\;}^{\circ }$C, the fully ordered L2${\;}_{1}$ structure transforms into the partly disordered B2 phase (CsCl-type), where the V and Al sublattices become disordered, that is, Al and V atoms can exchange and mix on the respective 4a and 4b Wyckoff positions. Then, at 1190 ${\;}^{\circ }$C, a further transformation into the fully disordered A2 structure (W-type) takes place. Maier et al. used in-situ neutron diffraction at high temperatures to investigate the structural transitions [115], and Garmroudi et al. modelled the order–disorder transitions using Monte Carlo simulations based on effective cluster interactions and statistical thermodynamics [34,116]. Moreover, in the Refs [34,116], it could be shown that the antisite defects present at high temperatures can be (partially) frozen by thermal quenching. Antisite defects involving the Fe sublattice lead to the formation of localized magnetic impurity states within or near the pseudogap of the compound. Thereby, the degree of antisite disorder can be directly correlated with the magnetic properties and even estimated from the saturation magnetization at low temperatures. At low concentrations, impurity states associated with these defects are localized due to Anderson and Mott localization and therefore do not contribute directly to thermoelectric transport. When increasing the concentration of defects, a quantum percolation threshold is reached, where electrons become delocalized in the center of the impurity band. This Anderson transition switches the conduction-type from p-type to n-type as charge carriers from the impurity states eventually outweigh holes from the valence band states in stoichiometric Fe_2_VAl [34]. Crucially, even though the system turns more metallic, the Seebeck coefficient increases and remains large as electronic states are stacked in a narrow energy interval, which enhances the density-of-states effective mass. This way, large values of the power factor up to 4 mW m${\;}^{-1}$ K${\;}^{-2}$ [35] and 7.6 mW m${\;}^{-1}$ K${\;}^{-2}$ [34] could be achieved in stoichiometric Fe_2_VAl via thermal treatment.

It is interesting to note that for stoichiometric Fe_2_VAl it is not possible to stabilize the fully disordered A2 phase by thermal quenching [34,107], whereas in Si-substituted Fe_2_VAl_0.9_Si_0.1_, the entire A2 structure can indeed be obtained experimentally in bulk samples, which was explained by a change in the diffusion kinetics of defects upon substituting Si for Al [116]. The A2 phase displays a ferromagnetic and fully metallic ground state, as demonstrated theoretically and experimentally [116,117]. Thus, an intermediate number of antisite defects is likely optimal for thermoelectric performance, although achieving reproducible samples and properties might be challenging, given the fact that ordering and diffusion kinetics during quenching change depending on the exact composition.

### 5.2. Inhibiting lattice-driven heat transport

Inarguably, the major bottleneck hindering the realization of high ZT values in Fe_2_VAl-based compounds is the intrinsically high lattice thermal conductivity of this compound. Depending on the exact synthesis conditions and the defects introduced thereby, $\kappa_{\mathrm{ph}}$ usually ranges between 15 and 30 W m${\;}^{-1}$ K${\;}^{-1}$ at 300 K, which is at least an order of magnitude higher than that of optimized Bi_2_Te_3_-based materials. Since heat carried by the lattice does not contribute to the conversion of thermal energy into electrical power, it reduces the efficiency of thermoelectric conversion processes and needs to be suppressed as much as possible. Owing to the high intrinsic $\kappa_{\mathrm{ph}}$ in Fe_2_VAl, provoked by very high values of the sound velocity, the primary focus of research has been on reducing $\kappa_{\mathrm{ph}}$ via various strategies. Below, we review some of the most promising conceptions that effectively reduced $\kappa_{\mathrm{ph}}$, and discuss ongoing challenges that need to be overcome to further boost ZT.

#### 5.2.1. Heavy-element substitution

**Figure 8.**
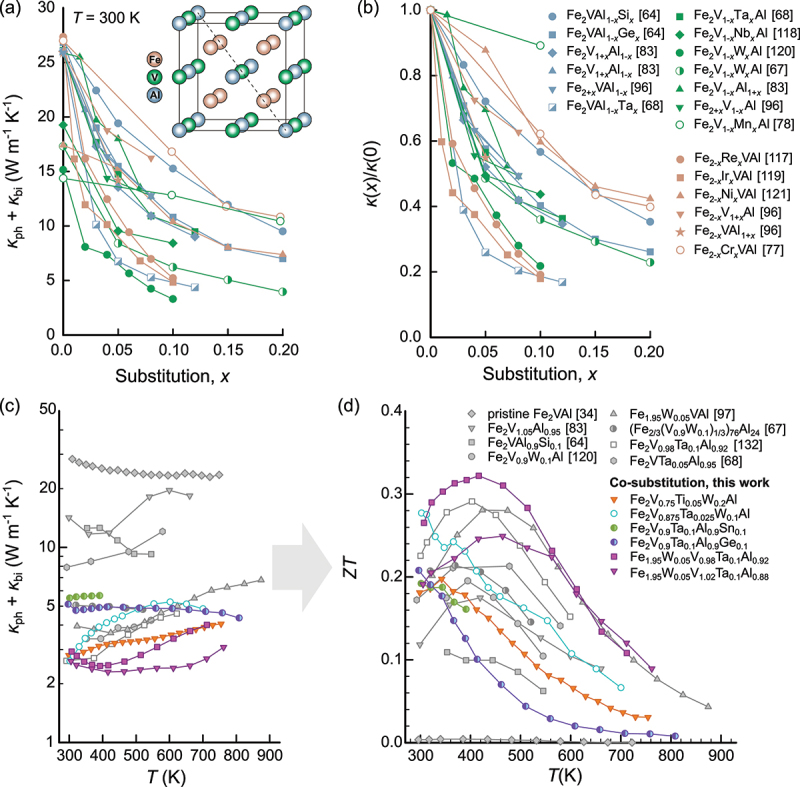
Reduction of lattice thermal conductivity by various substitutions [34,64,67,83,96,97,120,123,124] in Fe_2_VAl-based full-Heuslers. (a) Composition-dependent room-temperature lattice thermal conductivity plus bipolar contribution ($\kappa_{\mathrm{ph}}+\kappa_{\mathrm{bi}}$) of Fe_2_VAl-based systems with single-site substitution at Fe, V and Al sites. (b) Relative reduction of $\kappa_{\mathrm{ph}}+\kappa_{\mathrm{bi}}$ in (a) compared to the value of the stoichiometric compound reported in the respective literature study. (c) Temperature-dependent $\kappa_{\mathrm{ph}}+\kappa_{\mathrm{bi}}$ for different single-substituted and co-substituted and co-substituted Fe_2_VAl-based Heusler thermoelectrics. (d) Temperature-dependent ZT corresponding to the materials shown in (c). A record-high maximum ZT≈0.34 among all substituted systems is found for Fe_1.95_W_0.05_V_1.02_Ta_0.1_Al_0.92_ with substitution at all lattice sites. Gray symbols in (c) and (d) refer to data taken from the literature and colored symbols represent data collected for samples synthesized for the first time in this work. All materials were synthesized via induction melting as described in ref [40].

One of the most effective methods to reduce $\kappa_{\mathrm{ph}}$ in thermoelectric materials is alloying elements with large volume or mass differences. The associated strain and mass fluctuations will scatter high-frequency heat-carrying phonons. Figure 8 displays the combined efforts of several research groups to reduce $\kappa_{\mathrm{ph}}$ by extrinsic (co)substitution with foreign atoms [64,67,68,118–122] as well as by self-substitution via off-stoichiometric sample preparation [83,96]. The full-Heusler crystal structure with similar fcc sublattices and a vast phase space of elemental compositions allows substitution at all lattice sites, offering a huge playground for tuning functional properties. Since bipolar conduction plays a significant role at room temperature, $\kappa_{\mathrm{ph}}$ extracted from the total measured κ subtracted by the electronic part, which is estimated by the Wiedemann-Franz law, still contains the bipolar term, i.e. $\kappa -\kappa_{\mathrm{el}}=\kappa_{\mathrm{ph}}+\kappa_{\mathrm{bi}}$. Table 1(a, b) summarize the absolute values of $\kappa_{\mathrm{ph}}+\kappa_{\mathrm{bi}}$ and the relative reduction of $\kappa_{\mathrm{ph}}+\kappa_{\mathrm{bi}}$ with respect to pristine Fe_2_VAl as a function of the concentration of the substituted elements for different single-site substitution studies. It can be seen that substitutions with heavy 5d elements, such as Ta [121,125] and W [67,68] at the V site or Re [118] and Ir [120] at the Fe site, lead to the lowest values of $\kappa_{\mathrm{ph}}+\kappa_{\mathrm{bi}}$ with the exception of Fe_2_VAl_1_-xTa_x_. However, it was shown that, in this case, Ta preferentially occupies the V positions, and V atoms are pushed towards the Al site as antisite defects [121].

**Figure 9.**
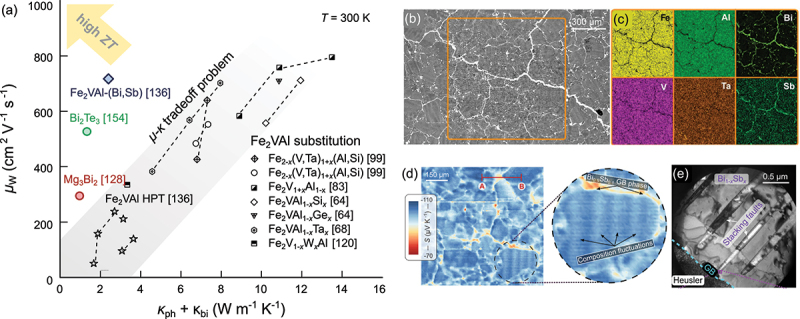
(a) Tradeoff between charge carrier mobility and lattice thermal conductivity. Weighted mobilities calculated using the equation presented in ref [126]. are plotted versus $\kappa_{\mathrm{ph}}+\kappa_{\mathrm{bi}}$ for various Fe_2_VAl-based systems, where lattice-driven heat transport is inhibited by substitution [64,68,83,99,121] or grain refinement [127] state-of-the-art Bi_2_Te_3_ [128] and Mg_3_Bi_2_ [129] semiconductors are also shown for comparison. Effective strategies that aim to boost ZT of Fe_2_VAl full-Heusler thermoelectrics have to overcome the μ-κ tradeoff problem. A promising strategy might be engineering appropriate grain boundary phases that allow for a decoupling of charge and heat transport [127]. (b) Backscattered electron microscopy images and (c) corresponding energy-dispersive x-ray mapping of the microstructure of Fe_2_VAl-(BiSb) composite with decoupled charge and heat transport shown in (a). (d) Transient potential Seebeck microprobe imaging reveals a complex microstructure with composition fluctuations of the Heusler phase inside the grains and Bi-Sb grain boundary networks. (e) Bright-field transmission electron microscopy reveals arrays of stacking fault defects within the Bi-Sb grain boundary phases. Images were represented from ref [127].

By co-substituting multiple elements at different lattice sites (see Figure 8(c,d)), $\kappa_{\mathrm{ph}}+\kappa_{\mathrm{bi}}$ can even be further reduced down to 2–3 W m${\;}^{-1}$ K${\;}^{-1}$ in Fe_1_.95W_0.05_V_0.98_Ta_0.1_Al_0.92_, which reaches a record-high ZT>0.3 for substituted Fe_2_VAl-based systems without grain refinement. As shown in Figure 9, the tradeoff between lattice thermal conductivity and carrier mobility, as a major challenge in further enhancing ZT by substitution in Fe_2_VAl-based full-Heuslers, is identified. This is particularly true for n-type materials, where 5d elements are substituted at the V site. As previously established, the conduction band of Fe_2_VAl and related Heusler compounds has predominantly $e_{g}$ orbital character attributable to the atoms at the Y site. Thus, introducing disorder at the Y site by alloying results in strong random potential fluctuations that heavily scatter charge carriers, deteriorating carrier mobility. One possible way to overcome this would be substituting heavy 5d elements at the Fe site (instead of the V site) and pushing the Fermi level towards the optimal position by Si or Ge substitution at the Al site. As of now, there are no reports on co-substitution studies investigating, e.g. Re or Os substitution at the Fe site, in combination with Si or Ge substitution at the Al site.

#### 5.2.2. Grain boundary engineering

There have been various attempts to refine the grain structure of Fe_2_VAl-based thermoelectrics, e.g. via ball milling [130,131] or high-pressure torsion (HPT) [123,132]. These techniques can decrease the grain size down to the nanoscale and induce lattice defects such as dislocations. Grain boundaries and dislocations scatter heat-carrying phonons of different wave lengths than those affected by point defects. They are, therefore, especially promising in conjunction with heavy-element substitution to scatter the whole phonon spectrum and optimally inhibit lattice-driven heat transport. Mikami et al. undertook efforts to synthesize various substituted Fe_2_VAl-based Heusler alloys via mechanical alloying in a planetary ball mill, followed by consecutive spark plasma sintering of the fine-grained powder [68,130,131]. The powder after ball milling usually consists of micrometric particles, which are agglomerates of even smaller nanocrystallites d≈20nm. Although grain growth occurs during the sintering process, the lattice thermal conductivity can be substantially reduced this way.

Masuda et al. [132] and Fukuta et al. [123] introduced high-pressure torsion as a technique to further reduce $\kappa_{\mathrm{ph}}$ in Fe_2_VAl-based full-Heuslers. HPT utilizes high pressures of several gigapascals and torsional strain, resulting in severe plastic deformation, to introduce an abundance of local lattice defects at different length scales (micro- to nanoscale). Rogl et al. have demonstrated that HPT can boost ZT in skutterudites up to ZT≈2 [133–135], while only slight improvements were reported for half-Heusler compounds [136]. Employing HPT to Fe_2_V_0.98_Ta_0.1_Al_0.92_ full-Heusler compounds, Fukuta et al. recently reported record-high ZT values (among Fe_2_VAl-based systems) up to ZT=0.37 [123]. As part of a recent study, it was attempted to reproduce these results, following the same synthesis procedure; however, no enhancements of the dimensionless figure of merit were realized [127]. This indicates that setup-specific conditions and parameters during HPT are likely crucial for realizing good thermoelectric properties, complicating upscale production. Another challenge regarding grain refinement, using the techniques described above, is that full-Heusler compounds are prone to antisite disorder, even at room temperature, given that enough mechanical work is induced [107,115]. Indeed, both ball-milled and plastically deformed Fe_2_VAl-based Heusler compounds commonly display a fully disordered A2 phase (see Figure 1(d)) instead of the L2${\;}_{1}$ Heusler structure. This phase has a magnetic and metallic ground state [116,117]. Thus, additional annealing at high temperatures is required to recover the desired Heusler phase, which inevitably results in grain growth, and consequently an increase of the thermal conductivity, compared to the nanostructured samples without annealing [123]. In addition, the tradeoff with respect to carrier mobility (Figure 9) further hinders enhancements of ZT.

In a recent work, composites with topological-insulating Bi_1-xSb_x-based materials were shown to decouple charge and heat transport, bypassing this tradeoff [127]. In this approach, chemically and structurally distinct Bi_1-xSb_x was incorporated between Heusler grains during synthesis via liquid-phase sintering, resulting in compact composite materials with a rich and complex microstructure (cf. Figure 9(b–e)). Surprisingly, only the thermal conductivity was reduced, while electrical transport remained excellent and the thermoelectric properties were significantly enhanced – results that go beyond effective medium theory predictions [137–139].

We summarize several important thermoelectric material parameters of optimized Fe_2_VAl-based full-Heusler compounds in Table 2, namely the lattice (and bipolar) contribution to the thermal conductivity, the weighted mobility [126], an estimate of the thermal band gap via the Goldsmid-Sharp formula and the thermoelectric performance given by $ZT_{\max}$ and the average ZT in the temperature range 300–400 K, which is most important for harvesting low-grade waste heat. It can be seen that while ZT has long hovered around 0.1–0.2, combined efforts of composition tuning and microstructure engineering have now resulted in several materials with ZT well above 0.3 or even 0.4, meaning that the ZT around room temperature of Fe_2_VAl-based materials is no longer an order of magnitude smaller than that of commercially used Bi_2_Te_3_ systems but merely a factor of 2–3.

**Table 2.** Important thermoelectric parameters for several optimized Fe_2_VAl-based full-Heusler compounds: phonon thermal conductivity (including bipolar term), weighted carrier mobility, Goldsmid-Sharp band gap $E_{g}=2e|S_{\max}|T_{\max}$, maximum ZT and average ZT in the temperature range 300–400 K.

Heusler material	$\kappa_{\mathrm{ph}}$ (W/Km)	$\mu_{w}$ (cm${\;}^{2}$/Vs)	$E_{g}$ (meV)	$ZT_{\max}$, $ZT_{\mathrm{av}}$
Fe_2_VAl_0.9_Si_0.1_ [64]	14.7	679	39	0.09, 0.08
Fe_2_VAl_0.9_Ge_0.1_ [64]	10.4	709	39	0.13, 0.12
Fe_2_V_0.9_W_0.1_Al [68]	3.4	314	44	0.20, 0.17
Fe_2_VAl_0.5_Ta_0.05_ [121]	7.8	715	66	0.22, 0.20
Fe_1.95_W_0.05_VAl [97]	4.0	651	51	0.28, 0.23
Fe_2_V_0.98_Ta_0.1_Al_0.92_ [123]	2.6	598	50	0.29, 0.27
Fe_2_VAl_0.95_Ta_0.05_ HPT [132]	3.3	508	52	0.30, 0.26
Fe2V0.95Ta0.05Al0.9Si0.1 [160]	6.8	1024	49	0.30, 0.26
Fe1.95W0.05V0.98Ta0.1Al0.92	2.9	597	48	0.32, 0.30
Fe_2_V_0.98_Ta_0.1_Al_0.92_ HPT [123]	1.3	439	50	0.37, 0.33
Fe_2_V_0.95_Ta_0.1_Al_0.95_-Bi-Sb [127]	2.4	724	44	0.46, 0.40

## 6. Ruthenium-based full-Heuslers

### 6.1. Comparison of ruthenium- versus iron-based Heusler compounds

**Figure 10.**
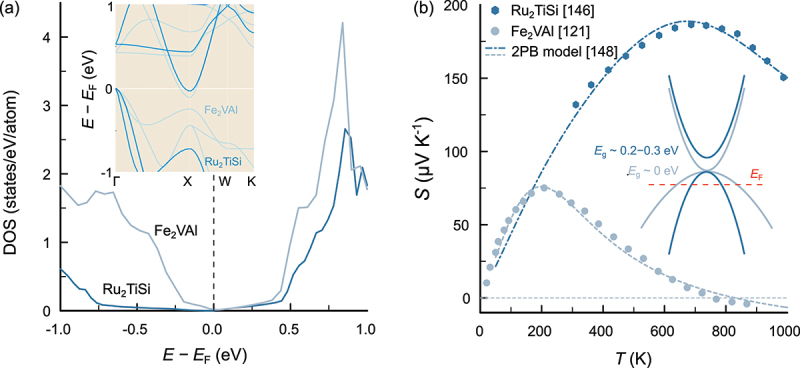
(a) Comparison of energy-dependent electronic density of states around the Fermi level for the stoichiometric full-Heusler compounds Fe_2_VAl and Ru_2_TiSi. Inset shows the band structures of both materials. More dispersive valence bands and a wider band gap is predicted for Ru_2_TiSi. Computational data were extracted from the materials project data base [148] (b) temperature-dependent Seebeck coefficient of Fe_2_VAl [122] Ru_2_TiSi [147] dashed and dashed-dotted lines represent least-squares fits, employing a two-parabolic band model (2PB). An analysis within a 2PB model [149] predicts a wider band gap and more dispersive valence band for Ru_2_TiSi, consistent with DFT calculations.

Among Fe-based full-Heusler compounds with VEC = 6, only Fe_2_VAl and Fe_2_VGa have been synthesized as single-phase materials. Although larger band gaps and semiconducting ground states have been predicted in other members such as Fe_2_TiSi and Fe_2_TiSn [140], Fe_2_NbAl [141] or Fe_2_TaZ (Z = Al, Ga, In) [142], these materials are either thermodynamically unstable compared to other more stable phases, in particular Fe_2_Y-based Laves phases [143], or could only be realized as metastable or disordered materials, e.g. employing thin film growth [144–146]. On the other hand, Fujimoto et al. recently reported thermoelectric properties of Ru_2_TiSi full-Heusler compounds. Due to the semiconducting ground state, the hitherto largest Seebeck coefficient among bulk full-Heusler systems was obtained over a broad range of temperatures [147]. In Figure 10, the electronic structures (extracted from the Materials Project database [148]) and the temperature-dependent Seebeck coefficient of Fe_2_VAl [122] and Ru_2_TiSi [147] are compared. DFT calculations of the density of states and band structure, shown in Figure 10(a), reveal that Ru_2_TiSi displays a wider pseudogap and more dispersive VB states compared to its isovalent sibling compound Fe_2_VAl. This can be interpreted as a result of increasing delocalization and bandwidth, when moving down the periodic table from 3d Fe towards 4d Ru. Indeed, the dispersion of the V/Ti-dominated conduction band at X hardly changes as opposed to the Fe/Ru-dominated valence band at Γ and X. An enlarged band gap and lower valence band effective mass, consistent with the DFT predictions, are also derived from least-squares fits of the temperature-dependent Seebeck coefficient S(T) of both compounds, employing a two-parabolic band model (see Figure 10(b)) [149]. Despite a similar slope of S(T) at low temperatures (implying a similar position of the Fermi level with respect to the VB edge), the maximum of S(T) is shifted towards higher temperatures for Ru_2_TiSi and reaches much larger absolute values. This is a clear indication of an enlarged band gap. Additionally, the tail of S(T) within the bipolar regime is less sharp, signifying that valence band states are more mobile than in Fe2VAl. This becomes even more apparent for p-doped Ru2TiSi_1-xAl_x, as discussed in Ref [149].

### 6.2. Superior p-type thermoelectric performance in Ru_2_TiSi semiconductors

**Figure 11.**
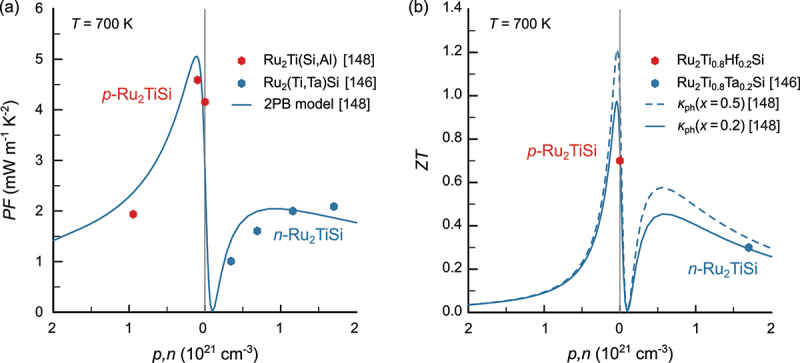
Modeling and thermoelectric performance predictions as a function of doping for Ru_2_TiSi-based Heusler compounds. (a) Doping-dependent power factor for p- and n-type Ru_2_TiSi. Solid line represents theoretical curve, calculated from a two-parabolic band model. Red symbols refer to experimental data of p-doped Ru_2_TiSi_1-x_Al_x_ from ref [149]. and blue symbols refer to n-doped Ru_2_Ti_1-x_Ta_x_Si from ref [147]. (b) Doping-dependent ZT. Solid and dashed lines were calculated for different values of the lattice thermal conductivity as discussed in ref [149]. Here, x refers to the concentration of heavy 5d elements substituted at the Ti site.

Fujimoto et al. studied the effect of Ta doping in n-type Ru_2_Ti_1_-xTa_x_Si [147]. Despite a significantly reduced lattice thermal conductivity, $ZT_{\max}\approx 0.4$ in Ru_2_Ti_0.8_Ta_0.2_Si, as reported by the authors, it did not exceed that of pristine Ru_2_TiSi, which also reaches $ZT_{\max}\approx 0.4$, suggesting that the weighted mobility of n-type samples is significantly smaller than for p-type compounds. This was confirmed in a previous study, where the temperature- and doping-dependent thermoelectric properties of both p- and n-type Ru_2_TiSi were analyzed, revealing that the valence band electronic structure displays a far greater potential for realizing high thermoelectric performance [149]. Figure 11 summarizes the doping-dependent prediction of the thermoelectric performance – PF and ZT – of Ru_2_TiSi semiconductors. For the latter, a similar reduction of $\kappa_{\mathrm{ph}}$ for different 5d elements substituted at the Ti site was assumed. Figure 11(b) shows that for a concentration of x=0.2 in Ru_2_Ti_1-x_Y_x_Si (Y = hf, Ta), a $ZT_{\max}$ close to $ZT_{\max}=1$ is predicted for p-doped Ru_2_TiSi, as opposed to a more than two times smaller $ZT_{\max}<0.5$ in n-type systems. These predictions motivated a study regarding the effect of Hf substitution in Ru_2_Ti_1-x_Hf_x_Si, for which $ZT_{\max}\approx 0.7$ is found when x=0.2 ,^1^ which likely can be further enhanced up to $ZT_{\max}\approx 1$ by co-doping Al for Si. These results demonstrate that, next to half-Heusler compounds, full-Heusler systems also bear the potential for realizing competitive thermoelectric performance, motivating a systematic search for novel semiconducting full-Heusler phases. Here, it should be pointed out that an increasing number of theoretical studies have predicted novel full-Heusler semiconductors X_2_YZ (X = Li, K, Ca, Sr, Ba; Y = Cs, Tl, Au, Ag; Z = Sn, Pb, As, Sb, Bi) with intrinsically ultralow thermal conductivities (owing to lattice anharmonicity) and extremely high ZT=2−5 [150–153]. These predictions spark excitement but have yet to be confirmed experimentally, as the high cost, chemical reactivity, toxicity, etc., pose challenges for experimental investigations.

## 7. Conclusion

Summarizing, there has been substantial progress in the development of full-Heusler compounds for thermoelectrics. Fe_2_VAl-based systems, discovered more than two decades ago [66,154], are attractive for their low cost, good recyclability and excellent mechanical and chemical long-term stability. While $ZT_{\max}$ of these systems has long hovered below 0.2, ZT values larger than 0.3 are no longer an exception, and recent studies with sophisticated nano- and microstructure engineering have even reported ZT values up to 0.4–0.5 around room-temperature, closing in on those of state-of-the-art materials, such as Bi_2_Te_3_ with ZT=0.8−1 at 300 K [128,129]. Apart from enhancements of the figure of merit, there has been huge progress in understanding the electronic structure and how to tune it with respect to thermoelectricity [31,51]. Novel concepts, such as selective localization of charge carriers [36], ultrahigh off-stoichiometry [101,103,108,110] to expand the band gap and induce resonant states or incorporating topological-insulating grain boundary phases to decouple charge and heat transport [127], have been developed and successfully applied. Another interesting direction that has been explored is to enhance the Seebeck coefficient through magnetic spin entropy, which works particularly well for Fe_2_VAl-based systems that are close to the magnetic instability and can be easily tuned towards a weakly magnetic state via doping [35,155]. A future challenge will be combining these strategies to optimally enhance both electronic and thermal transport properties. The recent discoveries of Ru_2_TiSi-based full-Heusler semiconductors are reviewed and discussed, as well. These materials intrinsically display promising electronic structure features, such as a narrow band gap $E_{g}=0.2-0.3$ eV, highly dispersive valence band states and a lower thermal conductivity compared to FeVAl-based compounds [147,149]. Multi-carrier transport modeling reveals a high potential figure of merit ZT>1 for Ru_2_TiSi-based systems upon proper reduction of the lattice thermal conductivity. Even though the high cost of Ru poses constraints for widespread applications and makes these materials less attractive from an economic point of view compared to their Fe_2_VAl relatives, these findings serve as a proof-of-concept that high ZT can be achieved in full-Heusler compounds and motivate a systematic search of other stable full-Heusler phases with semiconducting ground states.

## Note

1. Manuscript in preparation, Garmroudi F., Serhiienko I., Parzer M., Pustogow A., Mori T. and Bauer E. (2025).
